# Inhibition of high-voltage-activated calcium currents by acute hypoxia in cultured retinal ganglion cells

**DOI:** 10.3389/fncel.2023.1202083

**Published:** 2023-07-03

**Authors:** Hanna Dumanska, Mariia Telka, Nikolai Veselovsky

**Affiliations:** Department of Neuronal Network Physiology, Bogomoletz Institute of Physiology, National Academy of Science of Ukraine, Kyiv, Ukraine

**Keywords:** retinal ganglion cells, somatic calcium channels, high-voltage-activated calcium currents, acute hypoxia, reversible and irreversible inhibition

## Abstract

Hypoxia is a common factor of numerous ocular diseases that lead to dysfunctions and loss of retinal ganglion cells (RGCs) with subsequent vision loss. High-voltage-activated calcium channels are the main source of calcium entry into neurons. Their activity plays a central role in different signaling processes in health and diseases, such as enzyme activation, gene transcription, synaptic transmission, or the onset of cell death. This study aims to establish and evaluate the initial effect of the early stage of acute hypoxia on somatic HVA calcium currents in cultured RGCs. HVA calcium currents were recorded in RGCs using the whole-cell patch-clamp technique in the voltage-clamp mode. The fast local superfusion was used for a brief (up to 270 s) application of the hypoxic solution (pO_2_ < 5 mmHg). The switch from normoxic to hypoxic solutions and vice versa was less than 1 s. The HVA calcium channel activity was inhibited by acute hypoxia in 79% of RGCs (30 of 38 RGCs) in a strong voltage-dependent manner. The level of inhibition was independent of the duration of hypoxia or repeated applications. The hypoxia-induced inhibition of calcium currents had a strong correlation with the duration of hypoxia and showed the transition from reversible to irreversible at 75 s of hypoxia and longer. The results obtained are the first demonstration of the phenomena of HVA calcium current inhibition by acute hypoxia in RGCs and provide a conceptual framework for further research.

## 1. Introduction

Hypoxia in terms of its duration is divided into acute, sustained, cyclic, and many other types. Each type has different dynamics, molecular mechanisms, and effects on cells and tissues (Weir and Olschewski, 2006; Kaur et al., 2008; Saxena and Jolly, 2019; Liu et al., 2022). Several ocular diseases and sight-threatening disorders including glaucoma, diabetic retinopathy, optic nerve atrophy, central retinal artery occlusion, and ischemic central retinal vein thrombosis are accompanied by retinal hypoxia of different durations (Tezel and Wax, 2004; Arden and Sivaprasad, 2011; Chidlow et al., 2017; Lazzara et al., 2020). In the pathogenesis of these diseases, the common issue is the degeneration of retinal ganglion cells (RGCs) and their axons, leading to vision loss and blindness (Osborne et al., 2001; Kuehn et al., 2005; Munemasa and Kitaoka, 2013; Potilinski et al., 2020; Tezel, 2021). Retinal ganglion cells (RGCs) are the output neurons of the retina that collect, process, and transmit signals to the visual centers of the brain. Changes in electrophysiological characteristics and activity parameters of RGCs induced by sustained and long-lasting hypoxia were previously investigated (Kaur et al., 2008; Chidlow et al., 2017; Lee et al., 2022; Warwick et al., 2022). However, there is very limited information about the early effects induced by acute hypoxia in RGCs (Gross et al., 1999). Such an initial cellular response might be targeted to prevent further development of RGCs degeneration.

Among all cellular regulatory mechanisms, cytosolic calcium plays a key role in the regulation of retinal homeostasis (Shahulhameed et al., 2020). Voltage-gated calcium channels are the main transducers of membrane depolarization into intracellular calcium transients (Simms and Zamponi, 2014). These channels are transmembrane multiprotein complexes and are classified into high-voltage-activated (HVA) and low-voltage-activated (LVA) based on their voltage-dependent activation. HVA calcium channels are known to play a vital role in numerous physiological processes such as synaptic transmission, cell excitability, gene transcription, and initiation of several intracellular signaling events (Clapham, 2007; Catterall, 2011; Dolphin, 2021). The dysfunction of different types of HVA calcium channels appears to be involved in several neurological and psychiatric disorders (Zamponi, 2016). The effect of sustained hypoxia on intracellular calcium concentration in RGCs has been investigated in the modeling studies of glaucoma and optic neuritis (Yamada et al., 2006; Sasaki and Kaneko, 2007). Obviously, calcium plays a dual role in healthy and injured cells, and investigating the very early effects induced by acute hypoxia on HVA calcium channels might provide the necessary electrophysiological basis to increase their capacity for functional recovery and survival. In this study, we focused on identifying and evaluating the initial effect of acute hypoxia on somatic HVA calcium channels and their recovery during reoxygenation in cultured RGCs.

## 2. Materials and methods

All procedures involving animals were reviewed and approved by the Ethics Committee of Bogomoletz Institute of Physiology National Academy of Science of Ukraine.

### 2.1. Primary culture of retinal cells

Primary retinal cell cultures were prepared from Wistar rat pups of both sexes from postnatal day 0 to day 1, as previously described (Dumanska and Veselovsky, 2019). Briefly, after the decapitation and eye enucleation, retinal tissue was enzymatically and mechanically dissociated into single cells. Suspensions with cell densities of 10^4^ cells/cm^2^ were placed on the coverslip coated with poly-L-ornithine (Sigma-Aldrich) in Petri dishes. Furthermore, cells were maintained in the incubator in a humidified atmospheric air with ~21% O_2_ enriched by 5 ± 0.5% CO^2^ at 37 ± 0.5°C. We added 10 μM cytosine-α-D arabinofuranoside (AraC, Sigma, USA) on the fourth day of cultivation to suppress glia proliferation and completely replaced culture media on the fifth day. During cultivation, we partially replaced culture media every 4–5 days to maintain a stable nutrition supply. The culture medium was composed of Minimum Essential Medium Eagle with Hepes supplemented with 26 mM NaHCO_3_, 1.25% insulin (Insulin from bovine pancreas, Sigma-Aldrich), and 10% horse serum (Gibco).

### 2.2. Electrophysiological recordings

We recorded HVA calcium currents in RGCs using the whole-cell patch-clamp technique in voltage-clamp mode. The extracellular solution contained the following: NaCl, 130 mM; MgCl_2_, 2 mM; CaCl_2_, 2 mM; TEA-Cl, 20 mM; 4-aminopyridine, 3 mM; glucose, 15 mM, HEPES 20 mM, (Sigma-Aldrich); and pH 7.4 (by adding NaOH). We added 1 μM of tetrodotoxin (TTX) to the extracellular solution to block sodium currents. The patch pipettes with internal tip diameters 1.0–1.5 μm were filled with intracellular solution containing Cesium acetate, 90 mM; CsCl, 20 mM; TEA-Cl, 20 mM; MgCl_2_, 4 mM; Na_2_ATP, 3 mM; NaADP, 0.5 mM; NaGTP, 0.5 mM; EGTA, 10 mM; HEPES, 20 mM (Sigma-Aldrich); and pH 7.4 (by adding CsOH). After the gigaomic contact formation, the pipette capacitance was compensated by using compensation adjustment on the amplifier. We recorded HVA calcium currents in response to a rectangular voltage pulse of 200 ms stimuli from −70 to 0 mV. The current–voltage relationships were elicited from holding potential of −70 mV using 100 ms steps to test potentials over the range of −50 to +50 mV in 10 mV increments every 10 s. Potentials were corrected for a junction potential of −10 mV. In the experiments, we used Ca^2+^ as the charge carrier; therefore, to avoid calcium-dependent inactivation, the intervals between stimuli were set to 10–15 s.

We monitored the quality parameters of voltage clamping, such as leakage current (I_leak_) and the time constant of capacitive current (τ_cap_), using applications of short (10 ms) small-amplitude hyperpolarizing rectangular stimuli (−10 mV). We analyzed the obtained data if the I_leak_ and τ_cap_ values varied within 5% of the mean values. The average series resistance was 16.6 ± 3.2 MΩ and was not electronically compensated. All experiments were performed at room temperature of 20–24°C. All data were recorded and digitized at 10 kHz using an Axopatch-1D amplifier, DigiData 1322A, and Clampex 9.0 software (Axon Instruments).

### 2.3. Acute hypoxia *in vitro*

We mimicked acute hypoxia during electrophysiological recordings by applying the hypoxic solution to the recorded RGCs using the fast local superfusion technique (Veselovsky et al., 1996). This method allows us to control the speed and area of the application and quick exchange (<1 s) of multiple solutions. The hypoxic solution was obtained by saturating the extracellular solution with nitrogen for 20 min. The duration of the hypoxia application was 45–270 s.

### 2.4. Polarographic measurements

The concentration of oxygen in the extracellular solution was measured by the polarographic method using a platinum microelectrode and Ag–AgCl reference electrode. The indicator microelectrode was made from a borosilicate glass pipette, into the tip of which we soldered a platinum wire with a diameter of about 0.3 mm and a length of the open area of about 30–40 μm. We recorded the diffusion current at an applied potential of −700 mV. Such a potential corresponds to the middle of the diffusion plateau of the electrode polarogram. For the calibration of pO_2_, we used two points by recording the diffusion current in solutions with defined and fixed oxygen concentrations. First, to obtain the zero point, we used a saturated sodium sulfite solution (8 mM). For the second point, we recorded the diffusion current in the control normoxic solution that was equilibrated with air. The pO_2_ in the normoxic control solution was set in the range of 150–155 mmHg. After 20 min of saturation of extracellular solution with nitrogen, the pO_2_ value was <5 mmHg. All measurements of oxygen concentration were performed in a laminar flow of applied normoxic and hypoxic solutions.

### 2.5. Data analysis

Conductance was calculated as follows:


(1) $$\begin{array}{l} G=\frac{I_{peak}}{\left(V-V_{rev}\right)} \end{array}$$

where I_peak_ is the peak current at each test potential, V is the test potential, and V_rev_ is the reversal potential. The relative conductance (G/G_max_)–voltage (V) curves, hereinafter referred to as G–V curves, were plotted and fit using the Boltzmann equation:


(2) $$\begin{array}{l} \frac{G}{G_{\max}}=G_{\max}+\frac{\left(G_{\min}-G_{\max}\right)}{\left[1+e^{\left(V-V_{1/2}\right)/k}\right]}, \end{array}$$

where G_max_ is the maximal conductance, G_min_ is the minimal conductance, V is the test potential, V_1/2_ is the voltage at half-maximal conductance, and k is the slope factor.

To trace whether hypoxia-induced inhibition is associated with changes in current kinetics, we normalized currents during the control, hypoxia, and reoxygenation periods.

As the level of hypoxia-induced inhibition varied in a quite wide range, we calculated the recovery index (η) as follows:


(3) $$\begin{array}{l} \eta =\frac{I_{wash}-I_{N2}}{I_{control}-I_{N2}}, \end{array}$$

where I_control_, I_N2_, and I_wash_, are mean amplitudes of calcium currents during control, hypoxia, and reoxygenation, respectively.

We calculated the confidence interval (CI) for each hypoxia duration to verify the recovery index switch from reversible to irreversible. We defined the recovery process as reversible if the value 1 was within the calculated confidence interval.

### 2.6. Statistical analysis

We used Origin 8.5 Pro and Clampfit 9.0 for data analysis and graphic presentation. The normality of data was checked with the Shapiro-Wilk test, and the difference between two sets of values was determined using a two-sample *t*-test. Spearman's rank coefficient of correlation was used as a non-parametric measure of statistical dependence of ranking between two variables. The results of Spearman's rank correlation were presented in the text as correlation coefficient (r_s_), *p*-values (*p*), and number of cells (*n*). All data in the text were presented as mean ± SD.

## 3. Results

**Figure 1:**
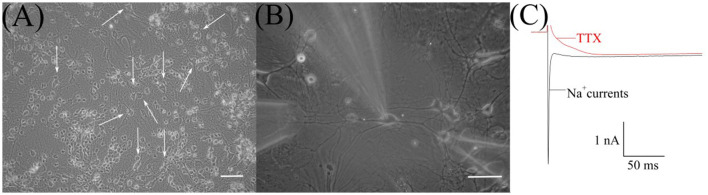
Microphotograph of cultured retinal ganglion cells (RGCs). **(A)** Primary culture of rat retinal cells on 19th day *in vitro*: white arrows indicate RGCs, scale marker corresponds to 100 μm. **(B)** Cultured RGCs during patch-clamp recording and fast local superfusion application on the 22nd day *in vitro*; the scale marker corresponds to 50 μm. **(C)** Representative recordings of sodium current before and after TTX application during electrophysiological identification of cultured RGCs.

In the culture, RGCs (Figure 1A) were easily identified initially by their morphological characteristics—soma diameters (Guenther et al., 1994). The diameters of RGC somata were 20–25 μm and calculated as the mean of the measurements by two perpendicular axes. Then, after establishing whole-cell configuration and before the HVA calcium currents recording, we confirmed RGCs identification by observing sodium currents with magnitudes ranging between 1 and 3 nA (Figures 1B, C). To observe sodium current, RGCs were first superfused with the normoxic extracellular solution without TTX. All further recordings in identified RGCs were performed in the presence of TTX.

**Figure 2:**
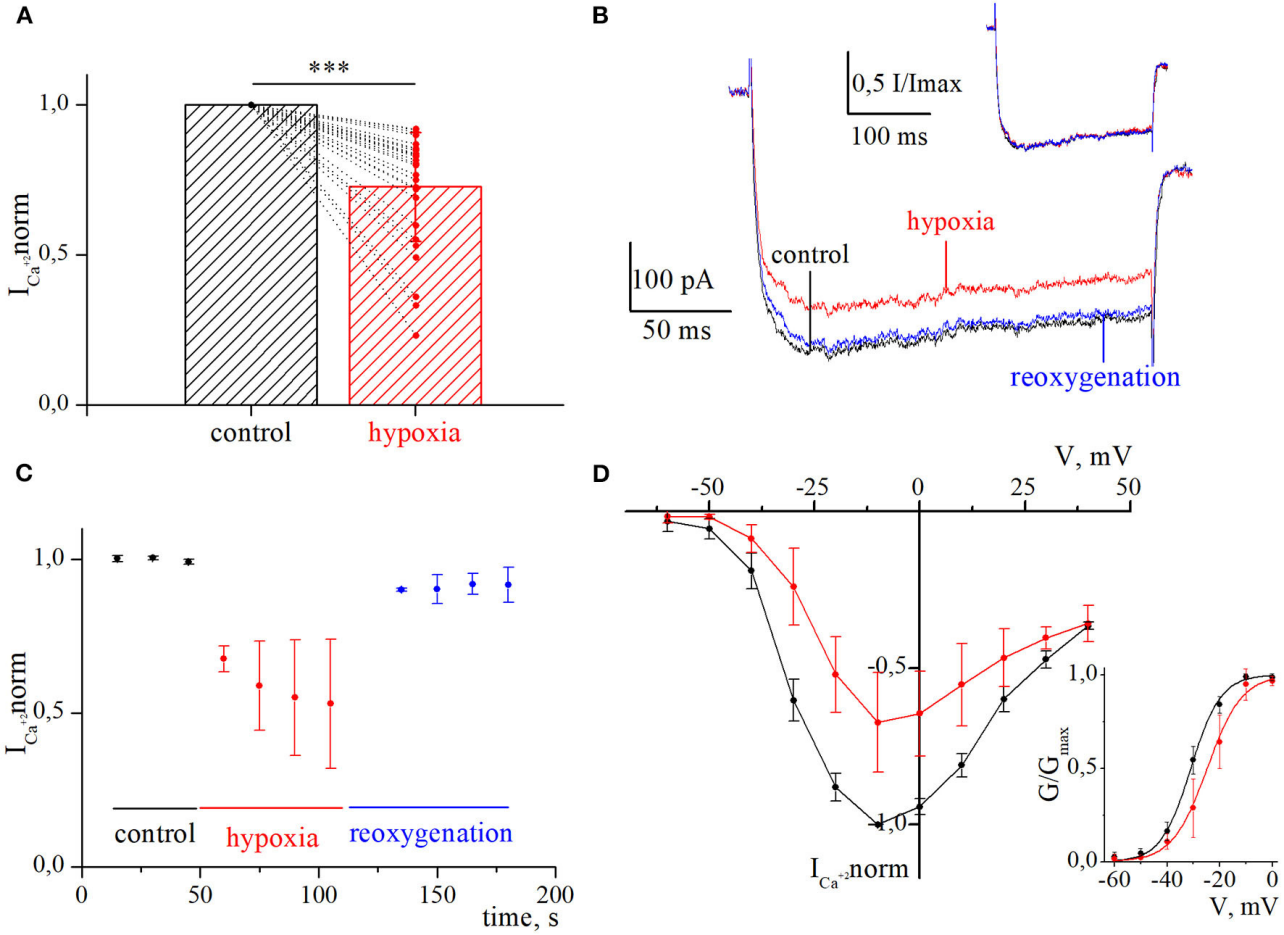
Inhibition of currents through high-voltage-activated calcium channels by acute hypoxia in retina ganglion cells. **(A)** The bar graph of the mean normalized values of calcium currents in control (black) and during the hypoxia (red); ***p < 0.001 compare to control. **(B)** Representative recordings of calcium currents under control, 60 s of hypoxia, and reoxygenation. The corresponding normalized currents are plotted above the recording. **(C)** The dynamic of averaged, normalized calcium currents amplitudes during control, 60 s of hypoxia (red), and reoxygenation (blue). **(D)** I–V relationships of averaged normalized calcium current amplitudes in control (black) and during hypoxia (red). The corresponding G–V curve of HVA calcium channels was built for control (black) and hypoxia (red). The solid lines are fitted with the Boltzmann curve.

We examined 38 RGCs from 11 to 32 days *in vitro*. All neurons exhibited HVA calcium currents with a peak magnitude ranging between 180 and 698 pA and the mean value is −316 ±137 pA, *n* = 38. The inactivation of currents developed extremely slowly. It was difficult to measure their time constants precisely because of the harmful effect of prolonged depolarization, but estimated values varied from 100 ms to 1 s. The I–V relationships of calcium currents have a clear single peak at a maintained potential of −10 mV. They do not have any low voltage-activated components, resulting in a plateau or peak at maintained potentials close to −40 mV (Figure 2D). Thus, the characteristics of HVA calcium currents in cultured RGCs were consistent with those previously described in the retina of the rat (Schmid and Guenther, 1999; Wanaverbecq et al., 2003).

We evaluated the initial effect of acute hypoxia on HVA calcium currents channels in cultured RGCs. The application of a hypoxic solution during 45–270 s led to the inhibition of HVA calcium current in most of the RGCs (79%, *n* = 30), in 10.5% of cells (*n* = 4) to augmentation, and 10.5% of RGCs (*n* = 4) remained insensitive to hypoxia. The evaluated level of effects induced by hypoxia of different durations and morphological and electrophysiological parameters of RGCs from these three distinct groups are shown in Table 1. Here, we did not observe any statistically significant differences in the parameters of recorded RGCs to classify them according to observed hypoxia-induced effects. The correlation analyses revealed that the level of inhibition was independent of the duration of hypoxia application (rs = −0.24, *p* = 0.2, *n* = 30), and the mean value was 27 ± 18% (varied from 8 to 77%; Figure 2A; Table 1). The representative recordings of inhibition of HVA calcium currents induced by 60 s of hypoxia are shown in Figure 2B. The normalized currents above the recordings represented the general tendency of hypoxia to decrease current amplitudes without any changes in current kinetics. The time course of HVA calcium current amplitudes showed that inhibition started to develop and reversed very quickly, within 15 s of application of the hypoxic and normoxic solutions, respectively (Figure 2C, *n* = 4). We also assessed the inhibitory effect of hypoxia over a broad range of membrane potentials and built an I–V relationship (Figure 2D; *n* = 3). Hypoxia-induced inhibition was strongly voltage dependent. The average hypoxic inhibition of HVA calcium currents was around 60% at −30 mV and 15% at +30 mV. Moreover, G–V plots revealed that hypoxia-induced a shift in the midpoint of the relative conductance curve to more depolarizing membrane potentials compared to control (V_1/2control_ = −31 ± 1.4 mV, V_1/2hypoxia_ = −24.8 ± 1.4 mV, *p* = 0.005). The slope factor remains unchanged (k_control_ = 5.7 ± 0.5, k_hypoxia_ = 6.4 ± 0.9, *p* = 0.53).

**Table 1:** Summary of the characteristics of hypoxia-induced effects on HVA calcium currents and corresponding electrophysiological parameters of RGCs.

**Hypoxia-induced effect on HVA Ca^+2^ currents**	**Inhibition**				**Augmentation**	**Insensitivity**
Hypoxia duration	40–50 s	60–70 s	75–85 s	≥100 s	40–270 s	40–270 s
Number of cells	*n* = 7	*n* = 7	*n* = 7	*n* = 9	*n* = 4	*n* = 4
Level of hypoxia-induced effect	27 ± 19	33 ± 15	21 ± 12	26 ± 18	15 ± 5	–
η	0.8 ± 0.16	0.69 ± 0.2	0.24 ± 0.16	0.04 ± 0.01		
	*n* = 7	*n* = 5	*n* = 4	*n* = 5		
	CI^99%^ 0.57–1.02	CI^99%^ 0.27–1.1	CI^99%^ −0.22 to 0.71	CI^99%^ 0.02–0.06		
I_Ca_, pA	−307 ± 109	−406 ± 194	−292 ± 109	−281 ± 114	−297 ± 154	−283 ± 65
R_in_, GOm	0.68 ± 0.32	0.77 ± 0.32	0.81 ± 0.41	0.7 ± 0.28	0.84 ± 0.36	0.86± 0.37
C_m_, pF	48 ± 13	56 ± 12	57 ± 13	50 ± 12	54 ± 12	57 ± 13
I_Na_, nA	~1.5	~1.8	~1.6	~1.7	~1.6	~1.8
Somata Ø, μm	23.3 ± 1.7	22.3 ± 1.9	22.8 ± 2	22.6 ± 2.2	22.8 ± 2.2	23.3 ± 1.7

The mean ± SD is given for each parameter. η, recovery index; CI^99%^, 99% confidence interval for given η; I_Ca_, magnitude of calcium current; R_in_, input resistance; C_m_, membrane capacity; I_Na_, magnitude of sodium current; Somata , diameter of somata.

**Figure 3:**
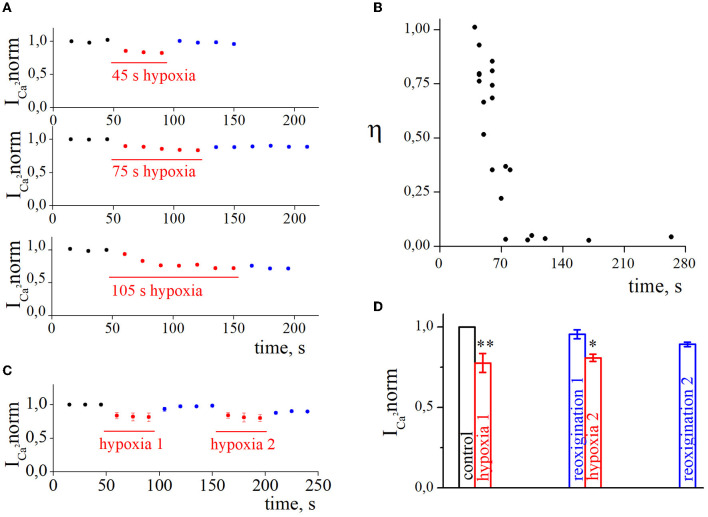
The recovery of high-voltage-activated calcium currents after acute hypoxia in retina ganglion cells. **(A)** The dynamic of normalized calcium currents amplitudes during control, 45 s/75 s/105 s of hypoxia (red), and reoxygenation (blue). **(B)** The dependence between the recovery index and the duration of hypoxia application. **(C)** The dynamic of averaged, normalized calcium currents amplitudes with repetitive 45 s of hypoxia application: control (black), hypoxia (red), reoxygenation (blue). **(D)** Bar graph of the mean normalized values of calcium currents with repetitive hypoxia application presented in **(C)**; *p < 0.05 **p < 0.01 compare to control.

We were interested in estimating not only the effect of hypoxia-induced inhibition of HVA calcium currents but also the recovery process during reoxygenation. The representative time courses of normalized HVA calcium current amplitudes with hypoxia application of different durations are shown in Figure 3A. As can be seen, the recovery during reoxygenation was dependent on the duration of hypoxia application, and the longer the hypoxia application, the less recovery during reoxygenation was observed. For quantitative evaluation of the recovery process and reversibility of hypoxia-induced inhibition, we calculated the recovery index (η). The dependency between the recovery index and the duration of hypoxia application (Figure 3B; Table 1) reflects the transition of inhibition from reversible to irreversible (r_s_ = −0.87, *p* = 8^*^10^−7^, *n* = 21). The inhibition of HVA calcium currents induced by 40–70 s of hypoxia was reversible, and the recovery index ranged between 0.53 and 1 with a mean value of 0.76 ± 0.2 (*n* = 12), whereas inhibition induced by 75 s of hypoxia or longer became irreversible, with the recovery index ranging between 0.03 and 0.36 with a mean value 0.13 ± 0.15 (*n* = 9).

We also tested the effect of repetitive application of hypoxia (Figures 3C, D, *n* = 4). The secondary application of hypoxia did not change the level of hypoxia inhibition (hypoxia_1_ 18.4 ± 2.1%; hypoxia_2_ 17.8 ± 1.1%; *p* = 0.6308) but decreased the recovery index during the second reoxygenation (reoxygenation_1_ 0.87 ± 0.03%; reoxygenation_2_ 0.4 ± 0.15%; *p* = 0.001).

## 4. Discussion

In this study, we investigated the initial effect of acute hypoxia (pO_2_ < 5 mmHg, up to 270 s) on somatic HVA calcium currents in cultured RGCs. In the majority of RGCs (79%), hypoxia led to the inhibition of HVA calcium currents. The level of inhibition was strongly voltage dependent but independent of the duration of hypoxia or repeated applications. The recovery index had a strong correlation with hypoxia duration and showed the transition of inhibition from reversible to irreversible.

To the best of our knowledge, this is the first demonstration of the phenomena of HVA calcium current inhibition by acute hypoxia in RGCs, which could contribute to the cellular compensatory response, preventing cells from hypoxic injury and improving neuronal survival (Kaur et al., 2008; Shimoda and Polak, 2011; Lange and Bainbridge, 2012).

The effect of calcium currents inhibition by hypoxia is not as commonly observed as hypoxia-induced augmentation. Hypoxia-induced inhibition of calcium currents is shown in taenia caeci smooth muscle cells (Rekalov et al., 1997) and in glomus cells (Montoro et al., 1996). Both these structures are involved in the oxygen-sensory body system due to their quick reaction to the direct action of hypoxia on oxygen-sensitive ion channels (Lahiri et al., 2005). The retina, as part of the visual system, has extremely high oxygen and energy demands (Cohen and Noell, 1965) and a unique O_2_ profile (Wangsa-Wirawan and Linsenmeier, 2003). It has been reported that the retina responds differently to acute and sustained hypoxia, from promoting blood flow to quick changes in transmembrane ion permeability and generation of vasoactive molecules to maintain oxygen homeostasis (Lange and Bainbridge, 2012). RGCs appeared to be particularly sensitive to different types of hypoxic stress (Kergoat et al., 2006), but what mechanisms mediate such a rapid reaction to changes in oxygen concentration is still unclear. For now, there is no confirmed evidence of the presence of oxygen-sensitive calcium channels in RGCs.

Previously, it has been shown that long-term hypoxia led to an increase in intracellular calcium ions concentration in RGCs (Yamada et al., 2006; Sasaki and Kaneko, 2007). Such an increase with subsequent cellular calcium overload can cause certain types of cellular death (Charriaut-Marlangue et al., 1996; Nicotera and Orrenius, 1998; Joo et al., 1999). On the other hand, brief periods of hypoxia, also called hypoxic preconditioning, can be adaptive for tissue and cells and show the neuroprotective effect on RGCs in a model of glaucoma and ischemic damage (Whitlock et al., 2005; Zhu et al., 2007, 2012, 2013). A strong dependence has been observed between hypoxia-induced effects on calcium channels and experimental conditions in terms of ionic compositions, duration of hypoxia, pO_2_ values and speed of its decrease, and the activity of voltage-dependent potassium channels (Montoro et al., 1996; Summers et al., 2000; Shkryl et al., 2001; Lukyanetz et al., 2003). Moreover, the activity of calcium currents by hypoxia might be modulated by several mechanisms. Hypoxia may lead to depolarization by the decrease of the potassium channels' activity (Montoro et al., 1996; López-Barneo et al., 2004); by ATP depletion (Hansen, 1985; Erecińska and Silver, 1994; Allen et al., 2005) and by the direct influence of hypoxia on calcium channels (Montoro et al., 1996; López-Barneo et al., 2004). Also, hypoxia-induced activation of NMDAR leads to an increase in intracellular calcium levels (Sucher et al., 1990; Siliprandi et al., 1992; Dumanska and Veselovsky, 2019).

In this study, we were able to record the initial effect induced by acute hypoxia, as the switch of pO_2_ from 150 to 5 mmHg was in <1 s during electrophysiological recordings of HVA calcium currents. According to our experimental conditions, detected inhibition of calcium currents by hypoxia was not dependent on changes in potassium channel activity, as these channels were blocked. Also, the hypoxia-induced inhibition was not associated with depolarization, as RGCs were voltage clamped. Furthermore, we observed that the inhibition of HVA calcium currents by acute hypoxia is strongly voltage dependent. Such a phenomenon has been previously observed in oxygen-sensitive potassium and calcium channels in glomus cells (Ganfornina and López-Barneo, 1992; Montoro et al., 1996) and arterial myocytes (Franco-Obregón et al., 1995). According to the literature, voltage dependence of hypoxia-induced inhibition is a characteristic feature of oxygen-sensitive channels. Moreover, the depolarizing shift of channel conductance, observed on the G–V plot, reflects that hypoxia-induced inhibition is associated with the reduction of voltage sensitivity of HVA calcium channels and the mechanisms of such a reduction should be investigated more closely. Whether calcium channels in RGCs might be oxygen-sensitive should be examined further, but all these together suggest the possibility that hypoxia directly inhibited calcium currents or was mediated by second messengers.

We also presented the transitions of hypoxia-induced inhibition of calcium currents from reversible to irreversible depending on the hypoxia duration. The inhibition appeared to become irreversible when the duration of hypoxia was 75 s or longer. It may reflect the involvement of different enzyme activation in time such as nitric oxide synthase (NOS) and nitric oxide (NO) synthesis. One of the NOS isoforms is calcium-independent iNOS. It has been reported that in hypoxic retina RGCs expressed iNOS and nNOS. The synthesized NO contributes to cytotoxicity, RGCs death, and axonal damage (Mishra et al., 2002; Zubrow et al., 2002; Kaur et al., 2006).

It is interesting that with the same parameters of hypoxia application, in 10.5% of recorded RGCs, we observed augmentation of HVA calcium currents and 10.5% of the cells remain insensitive to hypoxia. Such a diversity of responses to acute hypoxia might reflect the diversity of RGCs. There are various types of RGCs with specific gene expression, retinorecipient targets, morphological and physiological characteristics, and functions (Baden et al., 2021; Kim et al., 2021). Previous studies showed selective vulnerability of RGCs (Luo et al., 2009; Ou et al., 2016; Mayer et al., 2018) and type-specific survival and regeneration in response to different injuries (Tran et al., 2019; Tapia et al., 2022). Identification and classification of the general cellular mechanisms that either contribute to or inhibit type-specific RGC survival and regeneration are critical for the development of effective neuroprotective strategies.

We demonstrated that the diversity of hypoxia-induced effects in RGCs might also be associated with the type-specific composition of HVA calcium currents in different RGCs. Here we did not observe any difference in morphological or electrophysiological parameters of recorded RGCs to classify them according to observed hypoxia-induced effects, but the results obtained provide the platform for further research.

We also want to show that hypoxia, depending on its duration and magnitude, may cause a wide range of acid–base consequences such as alkalosis or acidosis (Swenson, 2016). In the presence of severe hypoxia, metabolic and hypercapnic acidosis develops with a high level of lactate formation and a fall in pH. Conventionally, acidosis is considered an additional injury that affects cell function and survival. However, sometimes it may also be cytoprotective to limit hypoxic injury. Mild extracellular acidosis, as a physiological consequence of oxygen deprivation, initiates mitochondrial metabolic pathway reprogramming to maintain the necessary level of ATP production in cortical neurons (Khacho et al., 2014). Some acidosis-induced effects were also shown in retinal cells. Activity-dependent drop in the pH, associated with exocytosis, inhibits HVA L-type calcium currents in bipolar cell terminals of goldfish retina (Palmer et al., 2003). Acidosis can also trigger a chain of intracellular processes, such as increasing the production of reactive oxygen species (ROS) by mitochondria (Riemann et al., 2011). It has been shown that ROS produced by RGCs' mitochondria modulate ion channel gating and excitability (Smith et al., 2023). It is also important to remember that RGCs can directly sense pH changes with TWIK-related acid-sensitive K+ (TASK) channels (Zhong et al., 2013; Wen et al., 2022) and to consider this in planning the research and interpretation of the results. In our research, we isolated hypoxia-induced effects using such a strong buffer as HEPES to maintain stable pH. In bicarbonate-based buffers, the hypoxia often leads to acidosis and it might cause a distortion of the investigating effects.

Summarizing the results obtained, we conclude that the inhibition of somatic HVA calcium currents in cultured RGCs induced by acute hypoxia reflects the initial compensatory reaction to the drop in oxygen level. It is not clear for now whether hypoxia affects calcium currents directly or due to the activation of some intracellular system. Taking into account the role of RGCs dysfunctions in blinding disorders, the mechanisms mediating detected inhibition should be investigated in further experiments.

## Data availability statement

The raw data supporting the conclusions of this article will be made available by the authors, without undue reservation.

## Ethics statement

The animal study was reviewed and approved by the Ethics Committee of Bogomoletz Institute of Physiology National Academy of Science of Ukraine.

## Author contributions

HD conceptualized the research ideas, prepared retinal cell cultures, and prepared the manuscript. MT designed and performed the electrophysiological experiments, analyzed data, and prepared the plots for further scientific interpretation. MT and HD contributed to research design creation, literature search, and interpretation of the obtained results with comments and suggestions from NV. All authors read and approved the final manuscript.
